# Phthisis

**Published:** 1889-10-26

**Authors:** 


### THE PRACTITIONER'S OUTLOOK AND RETROSPECT.

### MEDICINE.

### PHTHISIS.

Dr. A. Bowie contributes a paper "On the Inhalatory Treatment of Phthisis by means of Super-heated Air."* The author remarks that the bacilli of tubercle flourish only in a temperature of 99deg. If the temperature is lowered to 84deg., or raised to 107·6deg., they are rapidly killed. An encouraging case is reported under this treatment, and the author states improvement has resulted in several other instances. The apparatus used was Weigert's, in which the temperature expired by the patient, after a few trials, may reach 120deg. to 140. The inspired air, according to the thermometer in the tube, may be as hot as 570deg., but the author does not think the actual inspired air is ever hotter than from 280deg. to 300.

The inhalatory treatment of phthisis is as old as Hippocrates, and almost every conceivable method has been freely used with the exception of super-heated air. Phthisical patients have been recommended to spend a considerable portion of their lives in diving-bells to obtain the advantage of compressed air, and they have been recommended to go to high altitudes to obtain the advantage of less air pressure. We are not sanguine that phthisis will be cured by hot air inhalation.

